# Potential of *Bacillus subtilis* lipopeptides in anti-cancer I: induction of apoptosis and paraptosis and inhibition of autophagy in K562 cells

**DOI:** 10.1186/s13568-018-0606-3

**Published:** 2018-05-09

**Authors:** Haobin Zhao, Lu Yan, Xiaoguang Xu, Chunmei Jiang, Junling Shi, Yawen Zhang, Li Liu, Shuzhen Lei, Dongyan Shao, Qingsheng Huang

**Affiliations:** Key Laboratory for Space Bioscience and Biotechnology, School of Life Sciences, Northwestern Polytechnical University, 127 Youyi West Road, Xi’an, 710072 Shaanxi China

**Keywords:** Lipopeptide, Apoptosis, Anticancer, Paraptosis

## Abstract

The lipopeptide iturin from *Bacillus subtilis* has been found to have a potential inhibitory effect on breast cancer, alveolar adenocarcinoma, renal carcinoma, and colon adenocarcinoma. In this study, the potential of *B. subtilis* lipopeptides (a mixture of iturin homologues, concentration of 42.75%) to inhibit chronic myelogenous leukemia was evaluated using K562 myelogenous leukemia cells. The results showed that the lipopeptides could completely inhibit the growth of K562 at 100 μM, with an IC50 value of 65.76 μM. The lipopeptides inhibited the profile of K562 via three pathways: (1) induction of paraptosis indicated by the occurrence of cytoplasmic vacuoles, and swelling of the mitochondria and endoplasmic reticulum (ER) without membrane blebbing in the presence of a caspase inhibitor; (2) inhibition of autophagy progress illustrated by the upregulated expression of LCII and P62; and (3) induction of apoptosis by causing ROS burst, and induction of the intrinsic pathway indicated by the upregulated expression of cytochrome *c* (Cyto-c), *bax*, and *bad*, together with downregulated expression of *Bcl*-*2*. The ROS-dependent apoptosis and caspase-independent paraptosis were verified using the ROS inhibitor and caspase inhibitor, respectively. The extrinsic apoptosis pathway was not involved in the lipopeptide’s effects on K562. Overall, the *B. subtilis* lipopeptides (consisting of a majority of iturin) exhibited promising potential in inhibiting chronic myelogenous leukemia in vitro via simultaneously causing paraptosis, apoptosis, and inhibition of autophagy.

**Electronic supplementary material:**

The online version of this article (10.1186/s13568-018-0606-3) contains supplementary material, which is available to authorized users.

## Introduction

Chronic myelogenous leukemia (CML), also called chronic myeloid leukemia, is a cancer involving the bone marrow hematopoietic stem cells. It accounts for approximately 15% of adult leukemia (Shi et al. 2016). The typical properties of this disease are the chromosomal translocation and increased and unregulated growth of predominantly myeloid cells in the bone marrow, as well as the accumulation of these cells in the blood and spleen (Fu et al. 2016). Although stem cell transplantation (SCT) is an efficient method to cure CML, it tends to result in complications. Furthermore, it is very difficult to find a suitable donor for the patient (Kujak and Kolesar 2016). Tyrosine-kinase inhibitor (TKI) is another drug to treat CML and improve the long-term survival rate, greatly reduce pain, and improve the quality of life of patients. However, since it became a prominent anti-cancer drug in 2001, it has been found to be successful in only 5% of cases (Khan and Bixby 2014; Ross and Mgbemena 2014). TKIs can cause some side effects and certain toxicities, and patients may develop drug resistance or relapse due to chromosomal mutations, and these factors have limited its widespread use. Finding other efficient drugs is imperative to improve the cure rate for CML.

*Bacillus subtilis* lipopeptides have been found to have anti-tumor effects (Zhao et al. 2017), including inhibitory effects on human breast cancer in vitro and in vivo through the disruption of the Akt pathway (Dey et al. 2015), and the induction of apoptosis of melanoma A 375 cells by specific interaction with the plasma membrane (Janek et al. 2013) and human leukemia K562 cells associated with caspase-3 and poly(ADP-ribose)polymerase (PARP) protein (Wang et al. 2007). These lipopeptides are composed of a peptide ring and fatty acid chain and exhibit anti-bacterial, anti-inflammatory, anti-viral, and anti-tumor functions in vitro (Zhao et al. 2017). Some of them have been used as antiviral (Huang et al. 2006) and antitumor agents (Lee et al. 2012). Surfactin, iturin, and fengycin are the *Bacillus* lipopeptides that have been extensively reported because they possess antitumor activities. Surfactin can suppress the proliferation of the human colon carcinoma cell line LoVo (Kim et al. 2007), suppress TPA-induced breast cancer cell invasion through the inhibition of MMP-9 expression (Park et al. 2013b), and kill the human breast carcinoma cell line MCF-7 through a ROS/JNK-mediated mitochondrial/caspase pathway (Cao et al. 2010). Iturin inhibited the proliferation of breast cancer cells MDA-MB-231 (Dey et al. 2015) and MCF-7, alveolar adenocarcinoma A549, renal carcinoma A498, and colon adenocarcinoma HCT-15 (Hajare et al. 2013). Fengycin can block non-small cell lung cancer cell 95D and inhibit the growth of xenografted 95D cells in nude mice (Yin et al. 2013).

Currently, only a few studies are available on the ability of *B. subtilis* lipopeptides to inhibit chronic myeloid leukemia (Wang et al. 2007). However, surfactin has been found to have potential in curing blood diseases, such as preventing platelet aggregation and enhancing fibrinolysis with the facilitated diffusion of fibrinolytic agents (Lim et al. 2005). It was thought to have great advantages over other available thrombolytic agents in urgent thrombolytic therapy due to its fewer side effects and potential for long-term use.

In the present study, *B. subtilis* lipopeptides mainly being composed of iturin groups were used to treat K562 chronic myelogenous leukemia cells, and the intrinsic mechanisms were analyzed and explained at the gene and protein levels.

## Materials and methods

### Preparation of lipopeptide extracts

*Bacillus subtilis* CCTCCM207209, previously isolated from soil and stored at the China Center for Type Culture Collection (Wuhan, China), was used in this study to produce lipopeptides. *B. subtilis* lipopeptides were isolated from the supernatant of 48 h-culture in Nutrient Broth Medium with the inoculation amount of 2% and fermentation conditions of 32 °C, 160 rpm for 48 h (Inès and Dhouha 2015). The supernatant of the culture broth was collected by a 10-min centrifugation at 8000 rpm/min and 4 °C and filtered through a microporous filter membrane with pore diameter of 0.22 μm, followed by the adjustment of pH to 2.0 with HCl. The supernatant was stored overnight at 4 °C, and then the lipopeptide deposit was collected as a crude lipopeptide using centrifugation (8000 rpm/min, 4 °C, 10 min) (Coutte et al. 2015).

For further purification, the lipopeptide deposit was extracted using methanol, centrifuged (8000 rpm/min, 4 °C, 10 min), and then evaporated until almost completely dry. Then the lipopeptide was dissolved in deionized water for further purification using a Shimadzu LC-20A high efficiency liquid chromatography (HPLC) (Shimadzu, Japan) system quipped with a C18 column and detector at 280 and 215 nm. The mobile phase was a mixture of acetonitrile and water containing 0.1% trifluoroacetic acid with a flow velocity of 1.0 mL/min and a sample loading volume of 20–40 μL. The conditions for gradient elution were set as 0–40 min, 5–70% acetonitrile; 40–55 min, 70–100% acetonitrile; and 55–60 min, acetonitrile. Each fraction was separately collected and freeze-dried to obtain a powder that was further tested for its antitumor activity. The obtained powder was dissolved in pure water for the antitumor activity evaluation.

In order to identify the components of the obtained lipopeptide powder, each fraction separated from the powder was obtained using column chromatography and detected the antitumor activity. As results, the active fractions accounted for 42.75% of the total content of lipopeptides. As is shown in the Additional file 1: Figure: S1a, the peaks between the two red lines were the active fractions of lipopeptides, corresponding to seven different compounds in HPLC analysis. These active fractions were furtherly analyzed using electrospray ionization–high resolution mass spectrometry (ESI–MS) and identified as mainly iturin with molecular weight (*m/z*) from 1043.5 to 1065.5 Da according to the detected amino acid sequence (Additional file 1: Figure: S1b) and referring to the previously reports (Pathak and Keharia 2014). These fractions were diverse in the number of carbon atoms from 14 to 16 and similar in production. Such differences did not cause significant difference in the inhibition effect upon K562 cells. Therefore, in order to obtain an overview on the potential antitumor activity of *B. subtilis* lipopeptides, all fractions with antitumor activity were mixed together and used in the present study.

### Preparation and treatment of cell cultures

Human leukemia K562 cells was purchased from the cell bank of the Typical Culture Preservation Committee of the Chinese Academy of Sciences, and mycoplasma detection tests were performed on the cells to confirm that there was no occurrence of mycoplasma infection in the cells. The cultivation of cells was grown according to a previously reported method (Prayong et al. 2008) with slight modification. Specifically, the cells were grown at 37 °C and 5% CO_2_ in RPMI 1640 (Hyclone) medium that contained 1.5 g/L sodium bicarbonate and 4.5 g/L glucose supplemented with 10% fetal bovine serum (FBS).

For treatments, the purified lipopeptides were added to the culture of K562 cells at different concentrations of 6.25, 12.5, 25, 50, 100, and 200 µM (the concentrations were calculated using 1050 Da as the mean molecular weight of the mixed lipopeptide fractions). At different treatment periods from 0 to 60 h, the cell growth was detected to determine the cell proliferation, and other analyses were carried out at the same time.

### Cell proliferation analysis

The cell proliferation was tested using the Cell Counting Kit-8 (CCK-8, Dojindo, Japan) according to the manufacturer’s instructions with slight modification (Eike et al. 2015). K562 cells were seeded in 96-well plates at a density of 1 × 10^4^ cells per well with or without lipopeptide addition. At different growth periods, 10 μL CCK-8 (Sigma, USA) solution in PBS was added to each well. Plates were incubated for an additional 2 h, and the viability of the cultured cells was determined. After incubating the cells with CCK-8 solution, the light absorbance of the culture medium in each well was measured at 450 nm using a microplate reader (Bio-Tek, USA) (Prayong et al. 2008).

### Visualization of cell morphology

According to the IC50 (half maximal inhibitory concentration), the cells ready for morphology analysis were treated with 65.76 µM lipopeptide for 48 h. The treatment without lipopeptide addition was used as the control. The cells were seeded into 6-well plates at a density of 1 × 10^5^ cells per well. At the end of the treatment, the cells were visualized using a light microscope (Nikon 80i, Japan).

### Transmission electron microscopy

For electron microscopy analysis of cellular microstructure, treated and untreated K562 cells were fixed in 2.5% glutaraldehyde for 2 h at 4 °C, washed with Phosphate-Buffered Saline (PBS, pH 7.2) for 10 min three times, post fixed in 1% osmium tetroxide in PBS, washed with ddH_2_O for 10 min three times, dehydrated with gradient ethanol, rehydrated with epoxypropane, and subsequently embedded in epoxy resin. Ultrathin Sections (80 nm) were stained with uranyl and lead acetates and examined under a Hitachi H-600 (Hitachi, Japan) electron microscope at 80 kV.

### Mitochondrial membrane potential assay

The mitochondrial membrane potential (MMP) assay was carried out using a mitochondrial membrane potential assay kit with JC-1 (Beyotime, China), which undergoes potential-dependent accumulation in the mitochondria. In the assay, K562 cells were seeded into 96-well plates and treated with various concentrations of lipopeptides for 24 h, and then stained with 25 μM JC-1 for 30 min at 37 °C. After that, the cells were observed with a fluorescence microscope at the test wavelength of 540 nm (Lin et al. 2017).

### Detection of the occurrence of apoptosis

Apoptosis of the cells was detected using flow cytometry (BD FACSCalibur, USA) after staining with the Annexin V-FITC/PI Apoptosis Detection Kit (Beyotime, China). Cells stained only by annexin V are considered early apoptotic, and cells stained by both annexin V and PI are late apoptotic.

For the measurements, cells from either control or treated cultures were collected and washed twice with PBS (pH 7.2). Cells were resuspended in 195 µL of binding buffer, and then incubated with annexin V-FITC and PI for 15 min in the dark at room temperature. Finally, the analysis was performed with flow cytometry (BD FACSCalibur, USA) (Xu et al. 2017).

### Cell cycle assay

The distribution of the cells in the different phases was also measured using flow cytometry. PI was used to stain the DNA inside the cells. The portion of cells in different periods can be calculated according to the DNA content. After treatment with or without lipopeptides for 48 h, K562 cells were washed with PBS (pH 7.2) and fixed in 70% ethanol overnight at 4 °C. Then, PI (10 µg/mL) supplemented with RNase A (50 µg/mL) was added to the cells, which were incubated at 37 °C for 30 min. Analysis was performed with flow cytometry (BD FACSCalibur, USA).

### Visualization of apoptotic nuclear morphology

When apoptosis occurs, chromatin clumps and integrates, resulting in a crescent shape with margination, and the nuclear membrane disrupts. To visualize this, the lipopeptide-treated cells and untreated cells were washed with PBS three times, fixed with 4% paraformaldehyde for 10 min, and then stained with Hoechst 33342 (Solarbio, China), which was applied at a concentration of 5 µg/mL and then incubated for 10 min at room temperature. The observations were made with a Nikon 80i fluorescence microscope.

### TUNEL assay to determine the activity of endogenous nuclease

Endogenous nuclease is activated when cell apoptosis begins. Therefore, the DNA chain was cut to form 180 to 200 bp DNA fragments. The terminal deoxynucleotide transferase dUTP nick-end labeling (TUNEL) assay involves the specific addition of fluorescently labeled UTP to the 3′-end of the DNA fragments using terminal deoxynucleotidyl transferase. For measurements, the treated cells were washed with PBS twice, fixed in 4% paraformaldehyde for 20 min, and washed twice with PBS for 5 min each, at room temperature. The cells were then incubated at 4 °C for 5 min in citrate buffer (0.1% Triton X-100 in 0.1% sodium citrate), followed by washing twice with PBS at room temperature for 5 min each time. The TUNEL reagent (Beyotime, China) was prepared by mixing 5 µL of enzyme solution with 45 µL of labeling solution. Then, 50 µL of TUNEL reaction mixture was added per sample containing approximately 10^6^ cells. The mixture was incubated in the dark for 1 h at 37 °C. Then, the TUNEL reagent was removed, and the cells were washed twice with PBS, 5 min each time at room temperature. The washed cells were immediately viewed using a fluorescence microscope (Crowley et al. 2016).

### Measurement of reactive oxygen species (ROS)

The ROS level was measured using the dichlorodihydrofluorescein diacetate (DCFH-DA) assay. K562 cells were plated at a density of 1 × 10^5^/mL in 6-well plates. Then, 4 h later, lipopeptides were added for a final concentration of 65.76 µM, and the plates were maintained at 37 °C for 48 h. After that, the cells were stained with 10 µmol/L DCFH-DA (Beyotime, China) for 20 min at 37 °C. Then, the intensity of the fluorescence was examined with a multiwavelength multifunctional enzyme spectrometer (Synergy HT) and observed under a fluorescence microscope (Nikon 80i) (Yuan et al. 2014).

The ROS inhibitor *N*-acetyl-l-cysteine (NAC) was also applied to verify the ROS pathway. The cells were treated with NAC (2 mmol/L as a working concentration) for 1.5 h. Then, the cells were treated with the prepared lipopeptides. After the treatment, the ROS level was measured according to the referred method (Zhang et al. 2010).

### Western blot analysis

The cells treated with lipopeptides and the untreated cells were lysed with a homogenizer using RIPA lysis buffer with PMSF. The lysates were incubated on ice for 30 min with rotation. After 10 min of centrifugation at 12,000 rpm, the supernatants were collected, and the protein concentration was examined. Appropriate supernatants were boiled in loading buffer, separated by SDS-PAGE, and transferred onto a nitrocellulose membrane. Membranes were blocked with 5% (w/v) milk in TBST. Enhanced chemiluminescence (ECL) was used for immunodetection, and the procedure was performed as described in the ECL kit protocol (ServiceBio, China). Briefly, blots were incubated overnight at 4 °C with specific antibody, washed with TBST, and incubated for another 30 min at room temperature with the peroxidase-conjugated antibodies. Antibodies of Bcl-2, Bax, BAD, cytochrome *c* (Cyto-c), MEK1/2, JNK, LC3, P62, caspase-3, caspase-8, caspase-9, caspase-12, and β-actin were used in the assay.

### Cleaved caspase-3/9 activity assay

Caspase-3 activity was detected using a Caspase-3 Activity Assay Kit (Beyotime, China). Firstly, the cells were lysed in 100 µL lysis buffer for 15 min on ice. The lysates were then centrifuged at 16,000×*g* for 15 min at 4 °C. The supernatant of the cell lysates was mixed with buffer containing acetyl-Asp-Glu-Val-Asp *p*-nitroanilide (Ac-DEVD-pNA) as a substrate peptide and incubated at 37 °C for 3 h. The release of *p*-nitroanilide (pNA) was quantified by measuring the absorbance at 405 nm with a microplate reader (Bio-Tek, USA). The concentration of pNA was calculated from the pNA standard curve. One unit is the amount of enzyme that will cleave 1.0 nmol of the colorimetric substrate Ac-DEVD-pNA per hour at 37 °C under saturated substrate concentrations. The total protein concentration was measured by the Bradford method. The specific caspase-3 activity was normalized to the total protein and then expressed as a fold increase or decrease of the caspase-3 activity compared with the control group (Zhao et al. 2015).

To verify whether caspase-related apoptosis occurred, caspase inhibitor Z-VAD-FMK (methyl (3S)-5-fluoro-3-[[(2S)-2-[[(2S)-3-methyl-2-(phenylmethoxycarbonylamino) butanoyl]amino]propanoyl]amino]-4-oxopentanoate, Beyotime, China) was added to the cultured cells at a final concentration of 20 μM to inhibit the apoptosis pathway. After treatment, the cells were subjected to viability analysis using the CCK-8 assay, caspase-3/9 activity analysis, and TEM detection (Lee et al. 2016).

### Statistical analysis

All data were statistically analyzed using GraphPad Primer 6.0 (GraphPad Software Inc., San Diego, CA, USA). Significant difference analysis was performed using Student’s *t* test. The results are presented as the mean ± SD (standard deviation). The significance level was set at *P* < 0.05.

## Results

### *B. subtilis* lipopeptides inhibit the viability of K562

The effect of *B. subtilis* lipopeptides was analyzed using the CCK-8 assay. In this assay, the amount of yellow-colored formazan dye, generated by the activities of dehydrogenases in cells, is directly proportional to the number of living cells. The value of CCK-8 is related to the activity of dehydrogenases in cells. Dehydrogenases are stable enzymes, present in all cell types, and are rapidly released into the cell culture medium upon plasma membrane damage. The obtained OD value reflects the amount of residue of live cells with an intact plasma membrane, because the cells, instead of the liquid phase of cell culture, were used in the measurement.

**Fig. 1:**
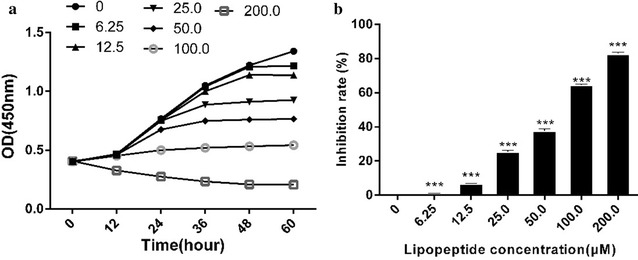
Effect of *B. subtilis* lipopeptides on the viability of K562. **a** The viability of K562 cells was measured at different periods; the concentration unit was μM. **b** The inhibition rate was calculated according to the data at 48 h; the significance analysis was performed on the data for treated and control samples using the software GraphPad Prism 6.0. (P < 0.01)

As shown in Fig. 1, the *B. subtilis* lipopeptides caused significant inhibition of K562 in a concentration dependent manner, as shown by the great decrease in the number of live cells (Fig. 1a, b). The OD value even decreased during the entire cultivation period when 200 μM of lipopeptides was used, indicating that the cells were damaged. The IC50 of the treatment for 48 h was 65.76 μM. These results indicated that K562 was sensitive to lipopeptides. Treatment with 65.76 μM (approximately 50 µg/L) of lipopeptides for 48 h was used in the following studies to illustrate the mechanisms that *B. subtilis* lipopeptides use to inhibit K562.

### *B. subtilis* lipopeptides inhibit the cell circle of K562

Some cells were completely damaged or inhibited by the lipopeptides, while Fig. 1a shows the activity of the live cell residue. These live cell residues warrant further examination, because it is unknown if they can maintain normal growth or if they will be disturbed in the growth cycle. In order to clarify this, we measured the cell cycles using flow cytometry.

**Fig. 2:**
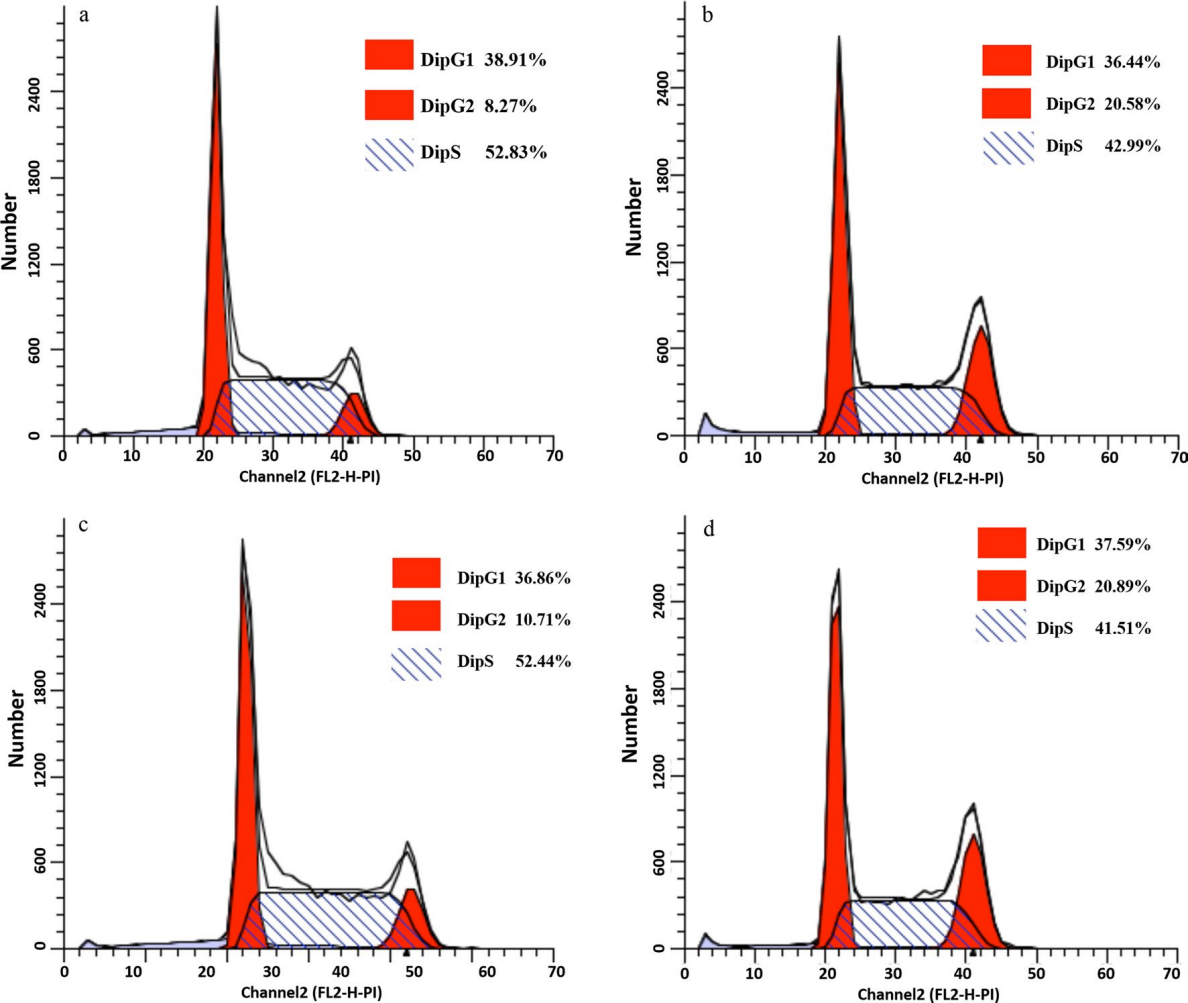
Flow cytometry analysis on the cell cycle of K562. In the treatment group, cells in S phase decreased significantly and increased in G2/M phase

Figure 2 shows the portion of cells in different periods, which was calculated according to the relationship between the DNA content and the intensity of the PI. The cells that were not treated with lipopeptides maintained relatively stable growth during the entire growth period, as indicated by the relatively stable portion of the cells in each period at 24 and 48 h (Fig. 2a, c). Compared with the untreated cells, the lipopeptide treatment resulted in an increase in the portion of the cells in G2 phase and a decrease in the cells in S phase at both 24 and 48 h. However, the portion of the cells in G1 phase was almost the same in both control and treated groups (Fig. 2b, d). This indicated that the lipopeptides did not disturb the supply of proteins, the increase of organelles (such as mitochondria, ribosomes), or the growth of cell size in G1 phase, but it inhibited the DNA replication in the S phase. The increase in the portion of G2 cells might be caused by the retardance of the cell from the prepared G2 phase to the M phase for cell division. Therefore, it can be deduced that the lipopeptides inhibited the growth of live K562 cells by blocking the DNA replication and the formation of microtubules and spindles.

There are three avenues to programmed cell death (PCD), and cancer cells can die as a result of any of these processes: apoptosis, paraptosis, and autophagy. As described above, the number of live cells was reduced by lipopeptides, and death might occur at the early stage of the treatment. These three PCD pathways were all examined in the following studies.

### *B. subtilis* lipopeptides induce the apoptosis of K562

Apoptosis is a programmed cell death that is normally regulated by genes and characterized by the occurrence of blebbing, cell shrinkage, release of apoptosis bodies, and the presence of nuclear fragmentation, chromatin condensation, DNA damage, and global mRNA decay. Once it has begun, the process of apoptosis cannot be stopped.

#### The formation of apoptosis bodies

**Fig. 3:**
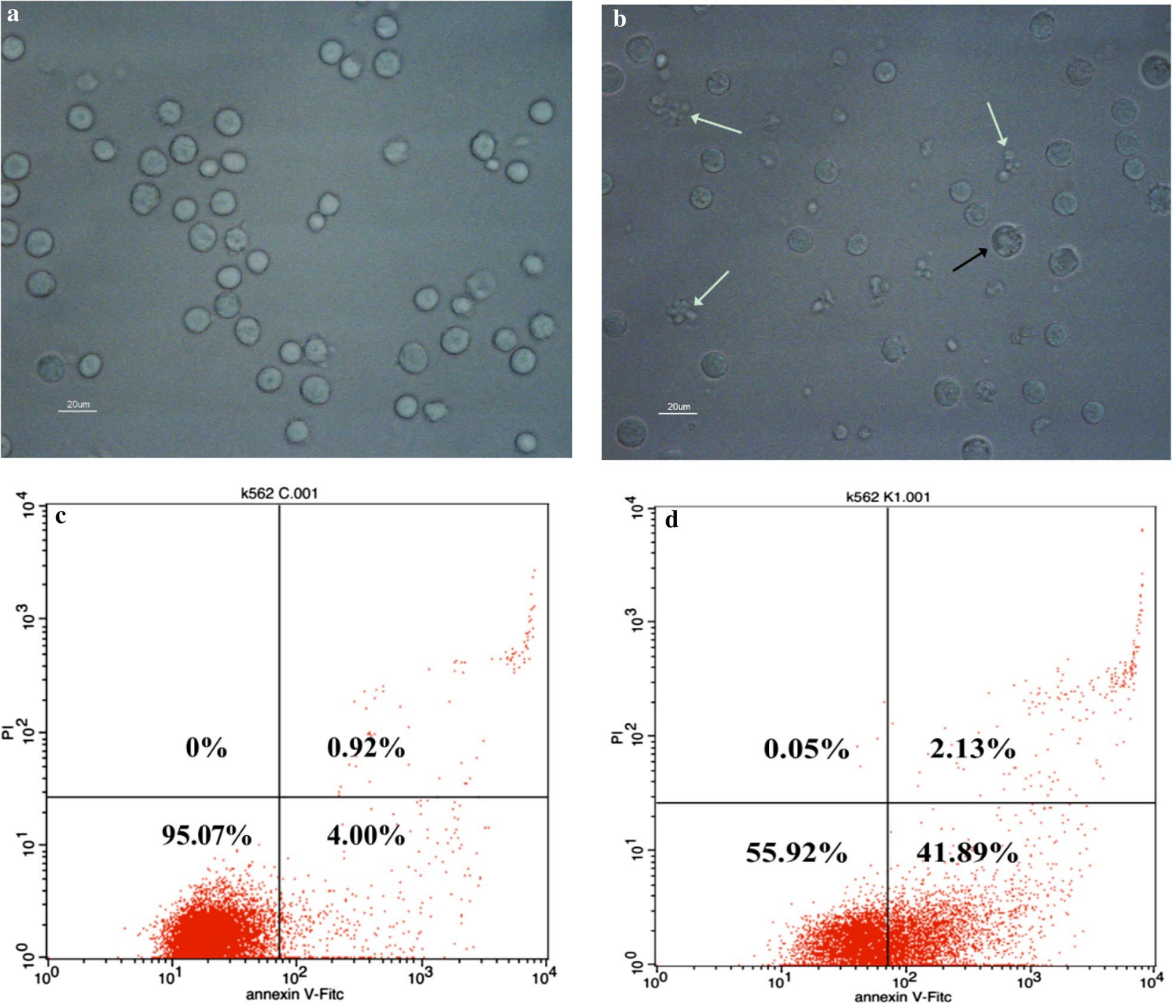
Micrographs and flow cytometry analysis of K562. The samples were measured after 48 h without (**a**, **c**) and with (**b**, **d**) treatment of 65.76 μM lipopeptides. **a**, **b** The white arrows indicate cells exhibiting the typical phenomena of apoptosis, with membrane blebbing, apoptotic membrane protrusions, and apoptosis bodies. The black arrow indicates the cells exhibiting paraptosis phenomena. The scale bar is 20 μm. **c**, **d** The proportion of apoptotic cells increased significantly

As shown in Fig. 3, after treatment for 48 h, an irregular surface, wrinkled edges, shrinkage and cleavage, and many apoptosis bodies were clearly observed in the lipopeptide-treated cells. As to be expected, the number of live cells in the lipopeptide-treated cells was less than that in the untreated cells in a visual field of similar size. All of these observations indicated that lipopeptide triggered the apoptosis of K562 cells. The observation of apoptotic bodies in the TEM photos of the lipopeptide-treated K562 cells also confirmed the induction of apoptosis (Fig. 9a).

It should be mentioned that the typical phenomena of paraptosis was also observed in a small portion of the cells after the lipopeptide treatment (Fig. 3b), and this was confirmed by the TEM photos (Fig. 9a).

#### The increase in the percentage of apoptotic cells

Figure 3c, d show the percentage of the cells at different status in the entire culture. The left lower part indicates the percentage of live and normal cells; the left upper part shows the cells in death; the right lower part presents the cells in the early stage of apoptosis; the right upper part shows the cells in the late stage of apoptosis. Compared with the untreated cells, the treatment with lipopeptides for 48 h caused a significant decrease in the percentage of live cells (from 95.07 to 55.92%), significant increase in the percentage of cells at the early apoptosis stage (from 4.00 to 41.59%), and a slight increase in the percentage of cells at the late apoptosis stage (from 0.92 to 2.13%). This illustrated that the *Bacillus* lipopeptides inhibited the viability of K562 by triggering abnormal apoptosis, and especially enhancing the occurrence of apoptosis at the early stage after treatment for 48 h.

#### The presence of chromatin condensation

**Fig. 4:**
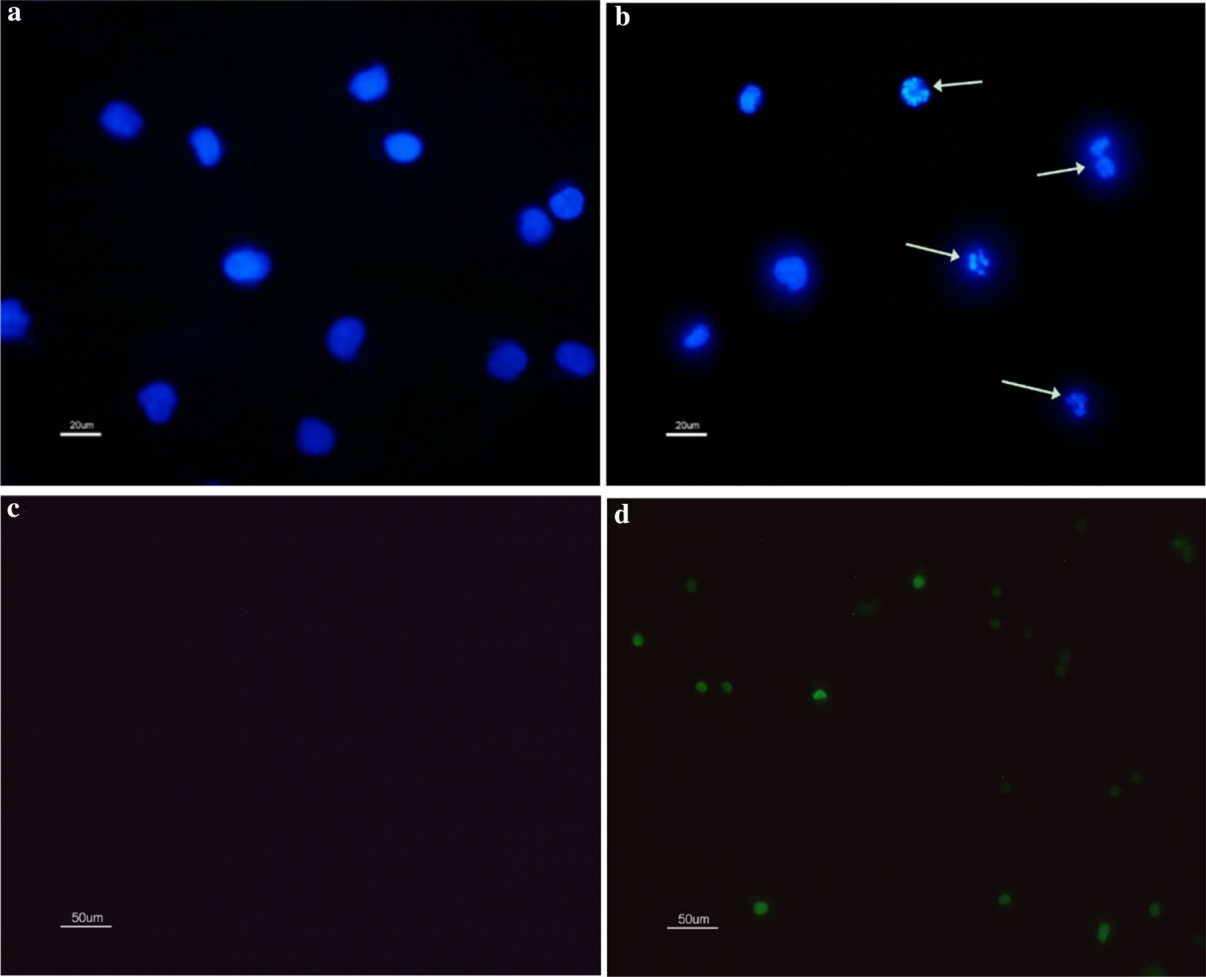
The nuclear morphology and DNA damage analysis of K562. The samples were measured after 48 h without (**a**, **c**) and with (**b**, **d**) treatment of 65.76 μM lipopeptides. **a**, **b** The white arrows indicate the condensed chromatin; The scale bar is 20 μm. **c**, **d** The green fluorescence indicates that the cell is apoptotic, and the intracellular DNase is activated to cut off the damaged DNA; The scale bar is 50 μm

Compared with the control, the treatment with lipopeptides reduced the number of live cells and caused the formation of condensed chromatin and the occurrence of nuclear lysis, which was indicated by the presence of condensed blue points of light inside the treated cells (Fig. 4a, b).

#### DNA damage

The TUNEL analysis showed enhanced green fluorescence density in the cells treated with *Bacillus* lipopeptides, indicating that the DNA in these cells was damaged (Fig. 4c, d). Therefore, it can be concluded that *B. subtilis* lipopeptides caused DNA damage by triggering the activity of DNase.

### Possible mechanisms for the induction of apoptosis in K562 cells

Apoptosis is characterized by the caspase-dependent pathways. One is the intrinsic pathway (also called the mitochondria pathway) characterized by the occurrence of mitochondrial swelling, increase in mitochondrial membrane permeability, and the leakage of apoptotic effectors, such as Cyto-c, mitochondrial apoptosis-induced channel (MAC), apoptotic protease activating factor-1 (Apaf-1), pro-caspase-9, caspase-9, caspase-3, Bcl-2, and Bax. Another one is the extrinsic pathway that involves mainly two theories: the TNF-induced (tumor necrosis factor) model and the Fas–Fas ligand-mediated model. The first one is characterized by the increased expression of TNF-alpha receptor (TNFR1); the second one has properties of enhanced expression of death domain (Fas-associated protein with death domain, FADD), caspase-8, and caspase-10. Among these caspases, caspases 8, 9, and 10 are initiator caspases that are activated through binding to specific proteins, while caspase-3 is an effector caspase that is subsequently activated by the active initiator caspases through proteolytic cleavage. Active caspase-3 proteolytically degrades the intracellular proteins to carry out the programmed cell death (Chen et al. 2014; Fulda 2011).

Some proteins and factors upstream of the intrinsic and extrinsic pathways also contribute to the occurrence and progress of apoptosis, such as JNK (Tran et al. 2007), extracellular signal-regulated kinases (ERK1/2), tumor protein p53 (Brown et al. 2009), and ROS. Compared with other well-known pathways, the roles of p53 and ROS in apoptosis have only been newly discovered. In the p53 pathway, upon DNA damage or other stresses, various pathways will lead to the dissociation of the p53 and mdm2 complex. Activated p53 binds DNA and activates expression of several genes including microRNA and inhibits their activity. ROS is normally produced in amounts that correspond to the occurrence of mitochondrial damage. It is newly reported that ROS can cause autophagy at low levels and apoptosis at high levels (Wu and Bratton 2013).

Herein, a series of studies was carried out at the level of morphologies and key gene expression to illustrate the mechanism for the apoptosis caused by the lipopeptides. The detailed results are below.

#### Decrease in mitochondrial membrane potential

**Fig. 5:**
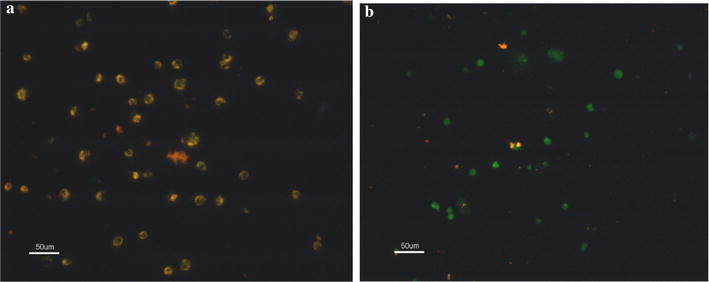
Mitochondrial membrane potential of (**a**) untreated and (**b**) treated K562 cells. Almost all cells in the control group showed more red fluorescence, while the lipopeptide-treated cells displayed green fluorescence. The scale bar is 50 μm

The monomeric JC-1 emitted green fluorescence, indicating a lower mitochondrial membrane potential (MMP). Aggregated JC-1 emitted red fluorescence, which implied a high mitochondrial membrane potential. It can be seen that the control cells mainly showed red fluorescence, while most cells in the *Bacillus* lipopeptides-treated group showed a significant increase in green fluorescence (Fig. 5). This indicates that the lipopeptides treatment decreased the MMP value of K562 cells to a low level, indicating that it was the lipopeptides that damaged the integrity of the mitochondrial membrane.

#### Increase in ROS level

**Fig. 6:**
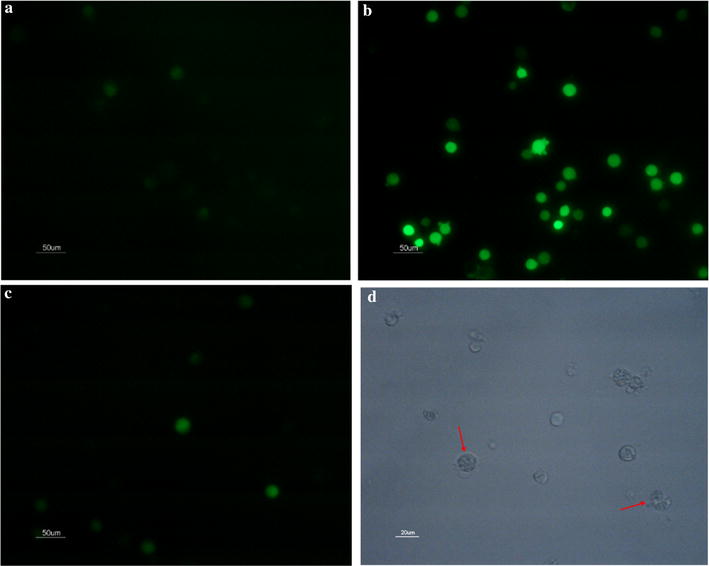
Reactive oxygen species of K562 cells. **a** The control cells without lipopeptide treatment; **b** the cells treated with 65.76 μM lipopeptides for 48 h; **c** the treated cells with ROS inhibitor; the scale bar is 50 μm. **a** The morphology of cells treated with lipopeptides and ROS inhibitors; the scale bar is 20 μm

Reactive oxygen species (ROS) are strong oxidizing substances in aerobic organisms and include oxygen radicals and their derivatives. It has been found that ROS is highly related to the occurrence of apoptosis. In the DCFH-DA assay, DCFH-DA can freely penetrate the cell membrane and be hydrolyzed by intracellular esterase to form DCFH after entering the cell. However, DCFH cannot permeate the cell membrane. Intracellular ROS can oxidize DCFH (without fluorescence) to DCF (with fluorescence). Thus, the level of intracellular ROS can be monitored by detecting the fluorescence of DCF. Figure 6 shows that the fluorescence of lipopeptides-treated cells was much stronger than that of the untreated cells.

**Fig. 7:**
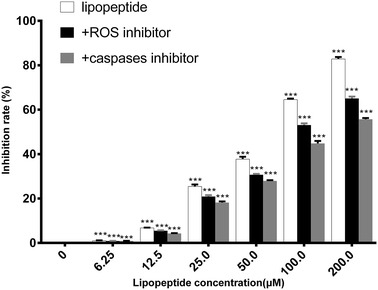
Influence of ROS inhibitor and caspases inhibitor on the inhibitory effect of lipopeptides on the viability of K562 cells. The data were obtained after the cells had been treated with lipopeptides for 48 h. When caspase enzyme activity and ROS accumulation were inhibited, the inhibition rate of K562 cells was significantly reduced

In order to verify the key roles of ROS in the induction of apoptosis, ROS inhibitor was used together with the lipopeptides. The presence of ROS inhibitor significantly reduced the occurrence of apoptosis and the inhibitory effect of *Bacillus* lipopeptides on the cell viability of K562 (Fig. 7). The cytoplasmic vacuoles still occurred when the cells were treated with lipopeptides in the presence of ROS inhibitor (Fig. 6d). These indicated that the induction of the enhancement of the ROS level played an important role in the mechanisms for the inhibitory effect of *B. subtilis* lipopeptides on K562, especially in the apoptosis pathway.

#### Expression of key proteins in apoptosis signaling pathways

**Fig. 8:**
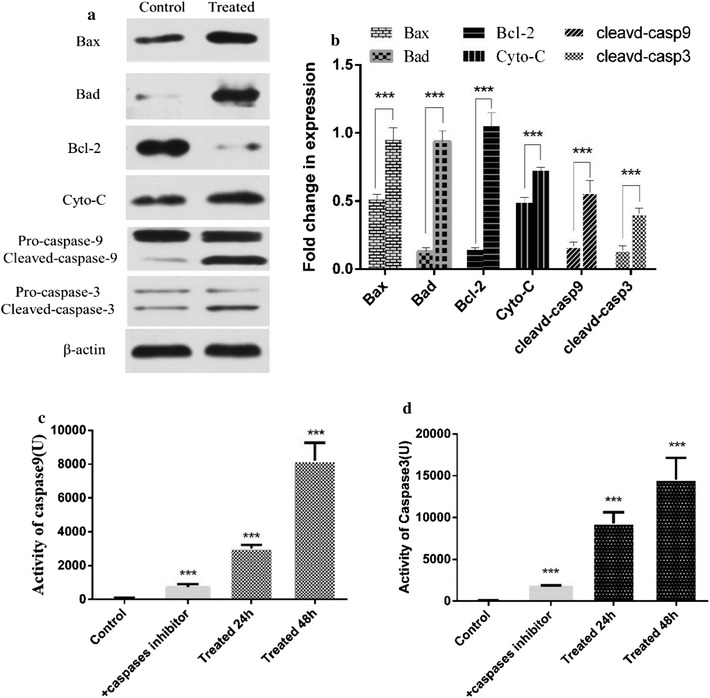
The expression of apoptosis-related key proteins and the activities of caspase-9 and caspase-3. **a**, **b** Western blot analysis was carried out after 48 h without (**a**) and with (**b**) treatment of 65.76 μM lipopeptide. **c**, **d** The activity was measured at different period with or without lipopeptide treatment. **c** the activity of caspase 9; **d** the activity of caspase 3. The caspase enzyme activity of the cells treated with lipopeptide increased as time went by. The caspase inhibitors effectively decreased the caspase enzyme activity

The following results were obtained according to the analysis shown in Fig. 8a, b and taking into account the currently reported apoptosis pathways. (1) The significantly increased expression of Cyto-c, Bax, Dad, and cleaved caspase-3, and the significantly decreased Bcl-2 indicates that the lipopeptides caused the apoptosis of K562 cells through the intrinsic pathway via the mitochondrial pathway. (2) The increased expression of cleaved caspase-9 and cleaved caspase-3 represents the activation of initiator caspases and effector caspases, respectively, indicating the detected extrinsic pathway. No significant change in p53, caspase-8, and caspase-12 indicates that the apoptosis caused by the lipopeptides was not related to these factors.

**Fig. 9:**
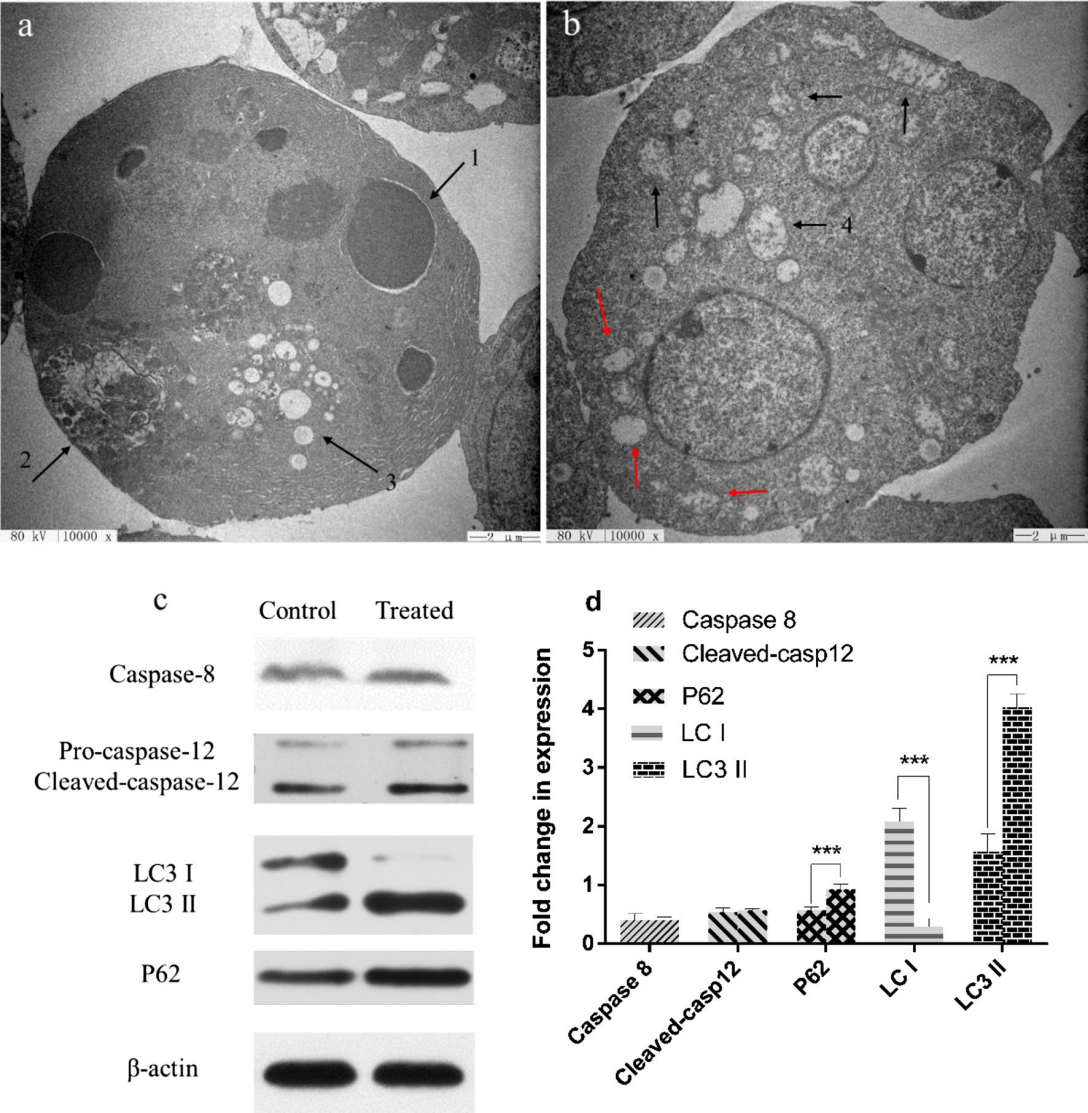
TEM photo of lipopeptide-treated K562 cells and the western blot analysis of the expression of apoptosis-related proteins. **a** TEM photo of lipopeptide-treated K562 cells. Arrow 1 shows the apoptotic body; arrow 2 indicates the autophagosome; and arrow 3 indicates the cytoplasmic vacuoles, typical markers of paraptosis. The scale bar is 2 μm. **b** TEM photo of K562 cells treated with lipopeptides together with caspase inhibitors. The black arrows mark the mitochondrion swelling, and the red arrows mark the ER swelling; the scale bar is 2 μm. **c** The expression of key proteins related to autophagy and paraptosis. **d** Quantitative analysis of the levels of gene expression shown in **c**

Furthermore, the enzymatic activity analysis showed that the activities of cleaved caspase-9 and cleaved caspase-3 were significantly increased by the *B. subtilis* lipopeptide treatment in a time- dependent manner (Fig. 8c, d). The presence of caspase inhibitor caused a significant decrease in the inhibitory effect of lipopeptides on the cell viability (Fig. 7) and inhibited the occurrence of apoptosis in K562 cells (Fig. 9).

Overall, it can be concluded that the *Bacillus* lipopeptides may cause the apoptosis of K562 cells through both the intrinsic pathway and one of the extrinsic pathways.

### *B. subtilis* lipopeptides inhibit the autophagy of K562

Cytoplasmic LC3 is commonly used as a marker of autophagosomes because it is the essential part of the vesicle and remains associated until the last moment before its fusion. At the occurrence of autophagy, LC3-I hydrolyzes a small polypeptide and turns itself into (autophagosome) membrane type LC3-II. P62 is another protein associated with autophagy, and it enters mature autophagy and is then degraded (Aburto et al. 2012). The lack of autophagy leads to the accumulation of p62. The increase in the ratio of LC3-II/I and the decrease in p62 are indicators for the development of autophagy. As shown in Fig. 9a, many autophagosomes were found inside the cells treated with the lipopeptides for 48 h. The western blot analysis showed that the treatment with lipopeptides upregulated the expression of LC3II and increased the LC3-II/I ratio, indicating that autophagy was induced. However, the accumulation of p62 was also increased in the lipopeptides-treated cells, indicating the lack of autophagy. This indicates that the autophagy process was inhibited by the lipopeptides and that the autophagy was eventually suppressed. Furthermore, this also indicated that the autophagy progress was inhibited at the stage of the combination of autophagosome with lysosome.

### *B. subtilis* lipopeptides induce the paraptosis of K562

Paraptosis is programmed cell death with a lack of apoptotic morphology, characterized by cytoplasmic vacuoles and swelling of the mitochondria and the endoplasmic reticulum (ER). This is similar to that of cell necrosis, but necrosis is usually accompanied by cell membrane blebbing. As is shown in Figs. 3 and 6, there are many cytoplasmic vacuoles in the lipopeptide-treated cells. This was more clearly indicated as the occurrence of cytoplasmic vacuoles, swelling of the mitochondria and ER, and the absence of apoptotic bodies and cell membrane blebbing when an apoptosis inhibitor was used together with the lipopeptides (Fig. 9b). This indicated that the formation of cytoplasmic vacuoles was independent of caspases. Moreover, the CCK-8 assay showed that the inhibitory effect of lipopeptides on K562 in the presence of apoptosis inhibitor could still be maintained at more than 50% of the original level (Fig. 7). This indicated that paraptosis might play an important role in the inhibitory effect of *Bacillus* lipopeptides on the cell viability of K562.

**Fig. 10:**
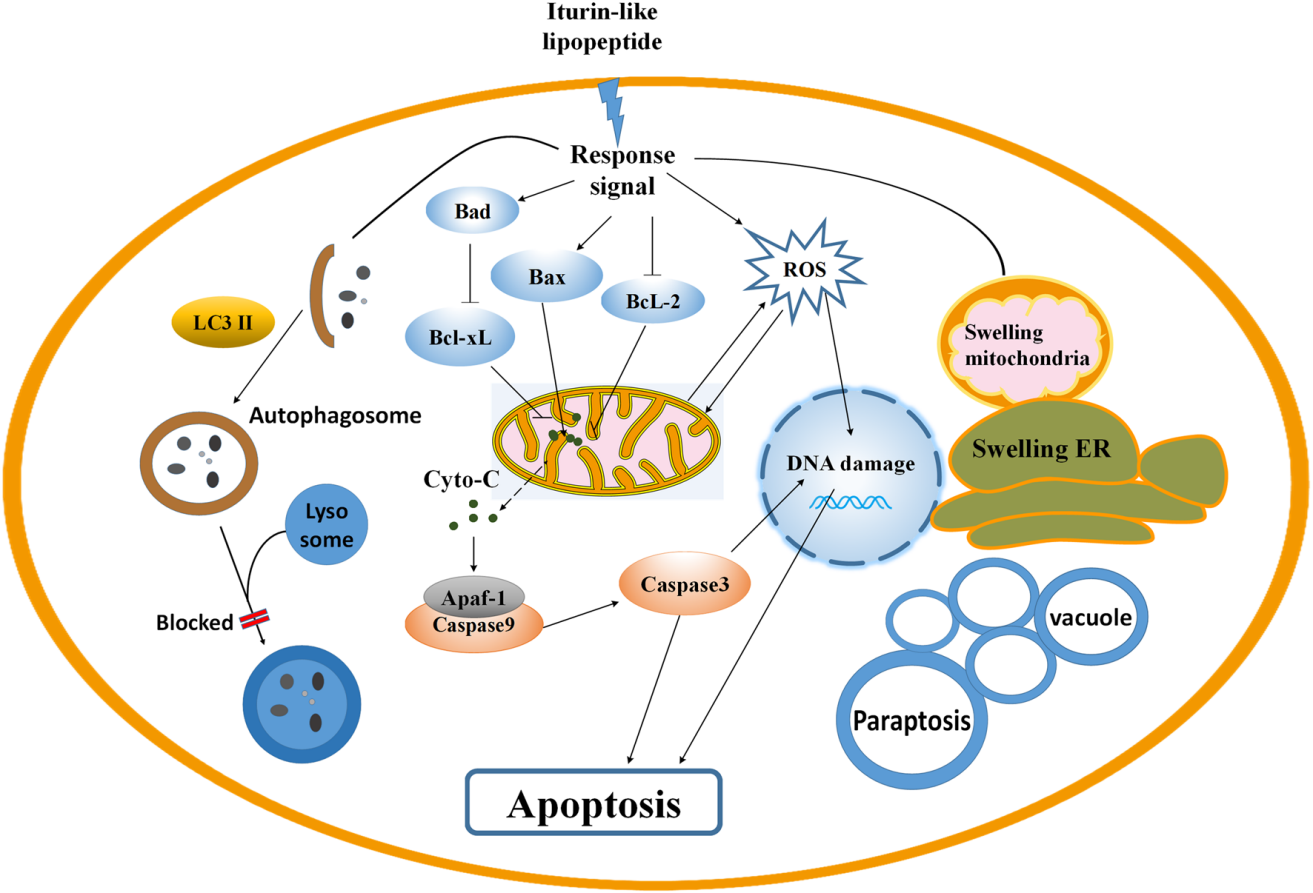
Overall mechanisms of the inhibitory effect of *Bacillus* lipopeptides on K562

Overall, the typical markers of paraptosis (the cytoplasmic vacuoles), apoptosis (the apoptotic body), and autophagy (the autophagosome) were all observed in the lipopeptides treated K562 (Fig. 9). The relative pathways were also illustrated in the view of key enzyme activities and the expression levels of key proteins and verified using the corresponding inhibitors. Therefore, it could be concluded that apoptosis, paraptosis, and autophagy occurred during the same period. The overall mechanisms for the effect of *Bacillus* lipopeptides on the cell viability of K562 was summarized in Fig. 10.

## Discussion

This is the first time to reveal the potential application of and the mechanism for iturin group in inhibiting blood cancer caused by K562. Many reports have indicated that *Bacillus* lipopeptides engage in anti-fungal, anti-bacterial, and anti-virus activities, and multiple therapeutic activities, such as anti-inflammation, anti-cancer, neuroprotective effects, inhibition of platelet aggregation, and immunomodulation therapy of diabetes (Zhao et al. 2017). The anti-cancer activities of *Bacillus* lipopeptides have been demonstrated with human breast cancer MCF-7 cells (Cao et al. 2010), and the human lung cancer cell line 95D (Yin et al. 2013). The current study is the first report describing the effect of *Bacillus* lipopeptides on blood cancer caused by K562 leukemia cells and indicates that *B. subtilis* lipopeptides, mainly the iturin groups, have potential in treating leukemia.

Further study is still needed to identify the exact structure of the active iturin fractions in this study. Lipopeptides produced by *B. subtilis* are a group of different molecular with similar molecular weight and structure. In this study, only 7 out of more than 30 lipopeptide fractions were found to have inhibitory effect on cancer cell K562. All of these seven fractions showed similar molecular weight and amino acid sequence as iturin. Therefore, it can be concluded that it was the iturin-like fractions in the *B. subtilis* lipopeptides to possess the anticancer potential, not all lipopeptide fractions. However, further study is still needed to identify the exact structure of these active iturin-like fractions. This work has been on the way in our lab.

Besides of induction of apoptosis in K562 reported by Wang et al. (2007), it was firstly revealed another two ways via by which *Bacillus* lipopeptides inhibit the viability of K562 cells: inhibiting autophagy progression and induction of paraptosis. Furthermore, the *Bacillus* lipopeptides used in this study was with m/z from 1043.5 to 1065.5 Da and identified as iturin groups, being different from that with m/z = 1072 Da in previous reports (Wang et al. 2007). In this way, this is the first time to reveal the capability of iturin groups produced by *B. subtilis* to show anticancer potential by inhibiting the viability of K562 cells.

The anticancer mechanisms of *Bacillus* lipopeptides have been extensively studied on surfactin, It was found to display an anti-proliferative effect via apoptosis induction, cell cycle arrest, and survival signaling suppression. Surfactin induces apoptosis in MCF-7 human breast cancer cells through a ROS/JNK-mediated mitochondrial/caspase pathway (Cao et al. 2010), suppresses TPA-induced breast cancer cell invasion through the inhibition of MMP-9 expression (Park et al. 2013a), and inhibits the growth of the human lung cancer cell line 95D through reactive oxygen species production and mitochondria-dependent apoptosis (Yin et al. 2013).

The reports of mechanisms for the anticancer effects of iturin and fengycin are comparably much less than those of surfactin. Fengycin interacts with lipid monolayers at the air-aqueous interface-implications on biological membranes. Iturin A inhibits Akt-mediated GSK3beta and FoxO3a signaling and triggers apoptosis in breast cancer (Dey et al. 2015). The induction of apoptosis by a mitochondria-dependent pathway has also been found in the mechanisms for the anti-fungal activities of *Bacillus* lipopeptides, including surfactin, fengycin, and iturin A. The current study revealed the inhibitory effect of *B. subtilis* lipopeptides consisting of a majority of iturin on K562 blood cancer cells.

Furthermore, the mechanisms for the apoptosis of K562 by *B.* *subtilis* lipopeptides were also extensively illustrated in the current study. Apoptosis is a widely reported mechanism for many anticancer agents (Ouyang et al. 2012; Wang et al. 1999). Lipopeptides have been found to kill many types of cancer cells by inhibiting ERK1/2 and Akt activation, causing ROS burst, cell-cycle arrest, enhancing the bax-to-bcl-2 expression ratio, and caspase activation (Cao et al. 2010; Hajare et al. 2013; Kim et al. 2007; Yin et al. 2013). However, no significant change was found in the expression of the extrinsic apoptosis pathway-related proteins of ERK1/2, Akt, JNK, Fas/FasL, and p53 (data not shown), indicating that these pathways were not found for the *Bacillus* lipopeptides tested in this study.

Some research indicates that ROS is involved in regulation of the intrinsic apoptosis pathway and plays important roles in the release of Cyto-c from mitochondria (Ricci et al. 2004; Wu and Bratton 2013). Additionally, ROS indirectly promote the formation of the apoptosome (Korytowski et al. 2011). Furthermore, Cyto-c release also disrupted the electron transport chain (ETC) and stimulated the production of ROS (Borutaite and Brown 2007). ROS were also found to be required for Fas-mediated apoptosome formation (Sato et al. 2004), and promote apoptosis via the extrinsic pathway through upregulation of death receptors or by serving as intermediates in the activation of kinases (Sinha et al. 2013; Tran et al. 2007). In this study, we also identified the important role of ROS in the inhibitory effect of the tested iturin-like  *B. subtilis* lipopeptides on K562. The role of ROS was also identified by the use of ROS inhibitor.

Inhibiting the autophagy progression of K562 was found as another mechanism for iturin groups to inhibit the viability of K562 cells. This was verified using an apoptosis inhibitor and measuring the expression of LC3II, p62, and the LC3-II/I ratio, which are important indicators of autophagy (Aburto et al. 2012). Other contributing factors were abnormal mitochondrial function or metabolic disorders caused by cytoplasmic vacuoles.

Furthermore, this study revealed that the iturin groups produced by *B.* *subtilis* could also induce the occurrence of paraptosis in K562. This is the first time that the capability of *B. sutbilis* lipopeptides to inhibit cancer cell growth by induction of paraptosis has been illustrated. Paraptosis is a programmed cell death that is different from apoptosis. The typical morphology consists of swelling of the mitochondria and endoplasmic reticulum, which may be attributed to the ion imbalances in the cells that cause excess water to move into the cell. The number and size of vacuoles increases over time. Eventually, the vacuole sizes reach a point of no return, and the cell cannot recover. The dysfunction of the mitochondria and endoplasmic reticulum accompanied with the disorder of metabolism eventually leads to the failure of the cells to survive (Lee et al. 2016), and these phenomena were observed in this study.

In conclusion, we proved that the lipopeptides (the majority consisting of iturin) from *B. subtilis* can completely kill K562 cells at a concentration of 100 μM, with an IC50 of 65.76 μM. Additionally, lipopeptides can cause ROS burst in K562 cells, promote the expression of *bax* and *bad* and inhibit the expression of Bcl-2, promote the release of Cyto-c, and cause apoptosis. The major effect pathway by which they operate is the endogenous apoptotic pathway. Moreover, the lipopeptides activated the autophagic flow but eventually suppressed it. At the same time, the lipopeptides induced paraptosis, resulting in a large number of cytoplasmic vacuoles, abnormal mitochondrial function, and disorder of cell metabolism. Although the effect of lipopeptides on K562 is obvious, further research is required before it can be used as a drug. Our research provides the initial data that can eventually be used for the treatment for leukemia.

## Additional file

**Additional file 1: Figure S1.** Chromatogram of purification (a) and ESI-MS (b) of major anticancer active lipopeptide fractions. **Figure S2.** Chromatogram of purification (a) and ESI-MS (b) of major anticancer active lipopeptide fractions.

## Abbreviations

CML: chronic myelogenous leukemia
SCT: stem cell transplantation
TKI: tyrosine-kinase inhibitor
HPLC: high efficiency liquid chromatography
CCK-8: Cell Counting Kit-8
IC50: half maximal inhibitory concentration
PBS: phosphate-buffered saline
MMP: mitochondrial membrane potential
PI: propidium iodide
TUNEL: the terminal deoxynucleotide transferase dUTP nick-end labeling
DCFH-DA: 2,7-Dichlorodi-hydrofluorescein diacetate
ROS: reactive oxygen species
RIPA: radio-immunoprecipitation assay
PMSF: phenylmethanesulfonyl fluoride
ECL: enhanced chemiluminescence
TBST: Tris Buffer Solution Tween
Bcl-2: B-cell lymphoma-2
Bax: Bcl-2 associated X Protein
Cyto-C: cytochrome c
MEK1/2: mitogen-activated protein kinase ½
JNK: c-Jun N-terminal kinase
LC3: microtubule-associated protein 1 light chain 3
Ac-DEVD-pNA: acetyl-Asp-Glu-Val-Asp *p*-nitroanilide
pNA: *p*-nitroanilide
Z-VAD-FMK: methyl (3S)-5-fluoro-3-[[(2S)-2-[[(2S)-3-methyl-2-(phenylmethoxycarbonylamino) butanoyl]amino]propanoyl]amino]-4-oxopentanoate
SD: standard deviation
PCD: programmed cell death
MAC: mitochondrial apoptosis-induced channel
Apaf-1: apoptotic protease activating factor-1
TNFR1: TNF-alpha receptor
FADD: Fas-associated protein with death domain
ERK1/2: extracellular signal-regulated kinases
MMP: mitochondrial membrane potential
