# Supplementary material for: Potential of Bacillus subtilis lipopeptides in anti-cancer I: induction of apoptosis and paraptosis and inhibition of autophagy in K562 cells
Source: AMB Express. 2018 May 9;8:78. doi: 10.1186/s13568-018-0606-3 (PMC5959823; doi:10.1186/s13568-018-0606-3)
Supplement: Supplementary file 1 — Additional file 1: Figure S1. Chromatogram of purification (a) and ESI-MS (b) of major anticancer active lipopeptide fractions. Figure S2. Chromatogram of purification (a) and ESI-MS (b) of major anticancer active lipopeptide fractions. [file 13568_2018_606_MOESM1_ESM.docx]

**Additional file**

**Potential of *Bacillus subtilis* lipopeptides in anti-cancer I: induction of apoptosis and paraptosis and inhibition of autophagy in K562 cells**

Haobin Zhao, Lu Yan, Xiaoguang Xu, Chunmei Jiang, Junling Shi ^*^, Yawen Zhang, Li Liu, Shuzhen Lei, Dongyan Shao, Qingsheng Huang

Key Laboratory for Space Bioscience and Biotechnology, School of Life Sciences, Northwestern Polytechnical University, 127 Youyi West Road, Xi’an, Shaanxi Province 710072, China

**Figure caption:**

**Figure S1 Chromatogram of purification (a) and ESI-MS (b) of major anticancer active lipopeptide fractions**

**Figure S2 Chromatogram of purification (a) and ESI-MS (b) of major anticancer active lipopeptide fractions**


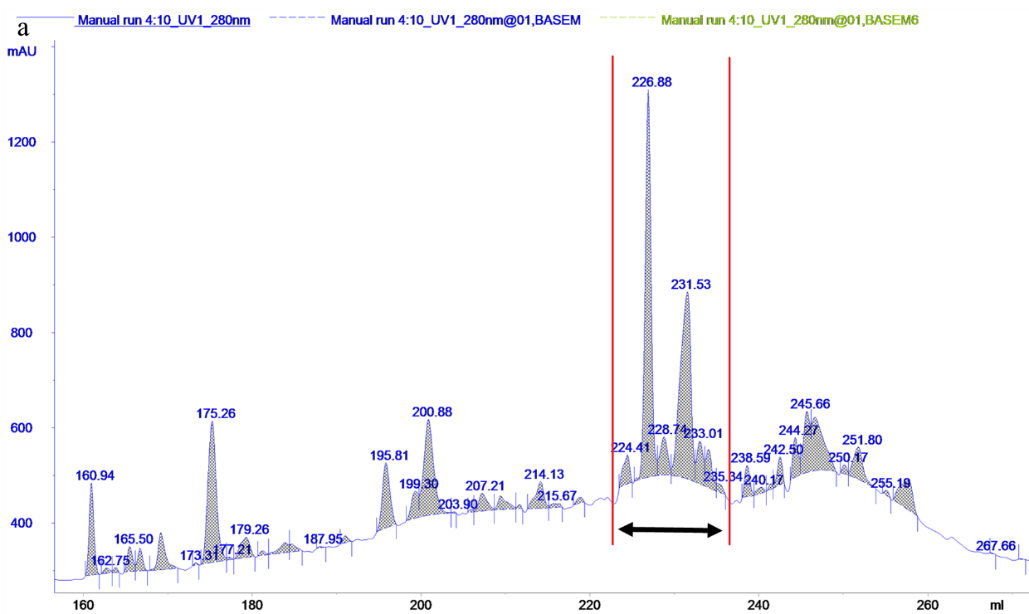

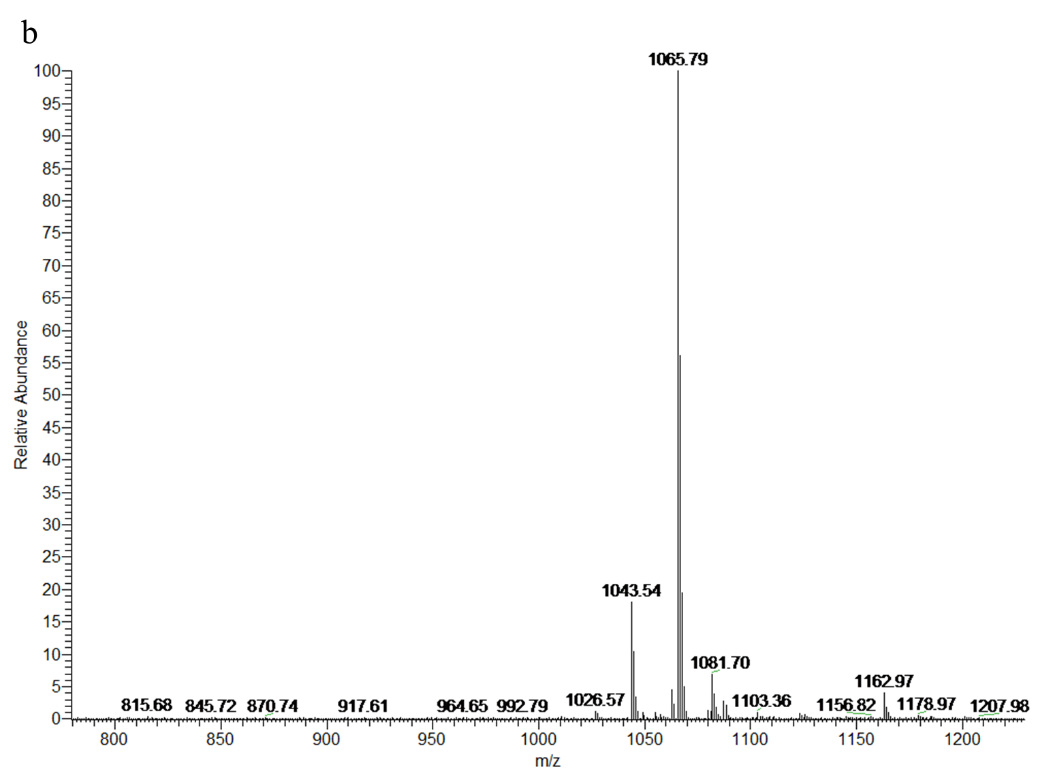


Figure S1

**
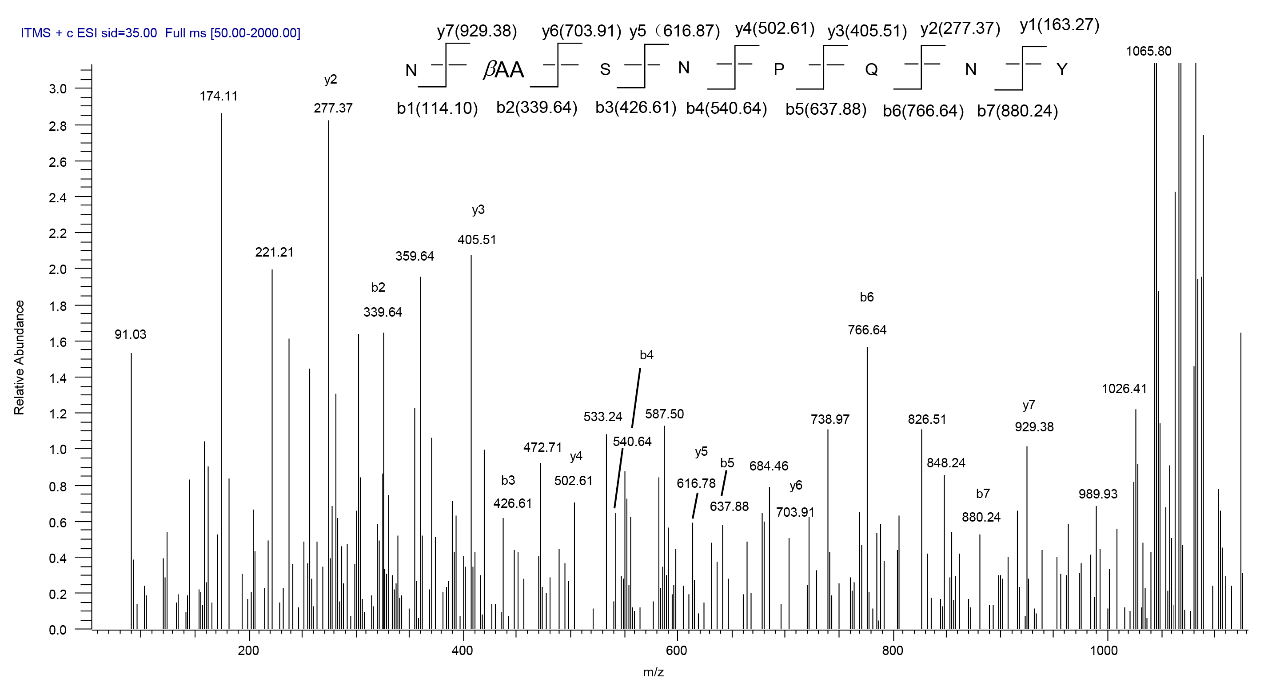
**

Figure S2
